# Identifying influential spreaders by gravity model considering multi-characteristics of nodes

**DOI:** 10.1038/s41598-022-14005-3

**Published:** 2022-06-14

**Authors:** Zhe Li, Xinyu Huang

**Affiliations:** 1 Software College, Shenyang University of Technology of China, Shenyang, 110870 People’s Republic of China; 2 Software College, Northeastern University of China, Shenyang, 110819 People’s Republic of China

**Keywords:** Complex networks, Physics

## Abstract

How to identify influential spreaders in complex networks is a topic of general interest in the field of network science. Therefore, it wins an increasing attention and many influential spreaders identification methods have been proposed so far. A significant number of experiments indicate that depending on a single characteristic of nodes to reliably identify influential spreaders is inadequate. As a result, a series of methods integrating multi-characteristics of nodes have been proposed. In this paper, we propose a gravity model that effectively integrates multi-characteristics of nodes. The number of neighbors, the influence of neighbors, the location of nodes, and the path information between nodes are all taken into consideration in our model. Compared with well-known state-of-the-art methods, empirical analyses of the Susceptible-Infected-Recovered (SIR) spreading dynamics on ten real networks suggest that our model generally performs best. Furthermore, the empirical results suggest that even if our model only considers the second-order neighborhood of nodes, it still performs very competitively.

## Introduction

The focus of network science has been shifting from discovering macroscopic statistical regularities to microscopic elements, vital nodes identification has received a huge amount of attention from researchers of network science in recent years. Vital nodes identification can be widely used in disease analysis^1,2^, rumor analysis^3^, information propagation^4^, power grid protection^5^, discovery of candidate drug targets and essential proteins^6^, discovery of important species^7,8^, and so on.

So far, most known methods only use structural information^9^, which can be classified into neighborhood-based centralities and path-based centralities roughly. Typical representatives of neighborhood-based centralities are degree centrality^10^ (DC), H-index^11^ and *k*-shell decomposition method^12^ (KS). For DC, the more neighbors a node has, the greater its influence. For H-index, the more large-degree neighbors a node has, the greater its influence. For KS, the more central a node locates in the network, the greater its influence. Besides, eigenvector centrality^13^ (EC) is the representative neighborhood-based iterative method, suggesting that the influence of a node is not only determined by the number of its neighbors, but also determined by the influence of each neighbor. Typical representatives of path-based centralities are betweenness centrality^14^ (BC) and closeness centrality^15^ (CC). For BC, the more a node is located in shortest paths, the greater its influence. For CC, the closer a node is to other nodes, the greater its influence.

However, a significant number of experiments indicate that depending on a single characteristic of nodes to reliably identify influential spreaders is inadequate^9^. As a result, the methods integrating multi-characteristics of nodes have been proposed. In particular, the methods based on gravity law seem very promising. As several laws behind phenomena in life are similar to the gravity law, the gravity model, which derives from the gravity law, is also favored and exhibited in many real-life scenarios. Representative examples include predicting the population migration between regions in demography^16^ and forecasting the trade flows throughout countries in economics^17^. In network science, the gravity model is utilized to evaluate the influence^18–20^ of nodes, and so on. Recently, a series of gravity-law-based algorithms^18–30^ considering both neighborhood information and path information have been proposed, and their performance is much better than the above well-known state-of-the-art methods. Typical representatives are gravity centrality^18^ (GC), improved gravity centrality^19^ (IGC) and local gravity model^20^ (LGM). For GC, the *k*-shell value of a node is regarded as its mass. For IGC, the focal node uses the *k*-shell value as its mass while its neighbors view the degree value as their masses. For LGM, the degree value of a node is regarded as its mass. However, whether the degree or *k*-shell is regarded as mass, the influence of neighbors is not taken into consideration. In view of this, we propose a gravity model that effectively integrates multi-characteristics of nodes to measure the influence of nodes in spreading dynamics. In our model, the number of neighbors, the influence of neighbors, the location of nodes, and the path information between nodes are all taken into consideration.

## Preliminaries

### Well-known state-of-the-art methods

Denote $G=<V,E>$ an undirected and unweighted simple network, where *V* and *E* are the sets of nodes and links. Denote $|V|=N$ and $|E|=M$, then the network has *N* nodes and *M* links. The adjacent matrix of *G* is denoted by $A=(a_{ij})_{N\times N}$, if node *i* links to node *j*, $a_{ij}=1$, otherwise, $a_{ij}=0$.

The degree centrality^10^ (DC) of node *i* is measured by

1 $$\begin{aligned} DC(i)=k(i), \end{aligned}$$

where $$k(i)=\sum _{j = 1}^{N} a_{ij}$$.

The H-index^11^ of node *i*, denoted by *H*(*i*), is defined as the maximal integer satisfying that there are at least *H*(*i*) neighbors of node *i* whose degrees are all greater than or equal to *H*(*i*).

The *k*-shell decomposition method^12^ (KS) works by iterative decomposition of the network into different shells. The first step of KS is to remove the nodes whose degrees are equal to 1 from the network, which will cause a reduction of the degree value to the remaining nodes. Continually remove all the nodes whose residual degrees are less than or equal to 1, until all the remaining nodes’ residual degrees are greater than 1. All the removed nodes in the first step form the 1-shell and their *k*-shell values are all equal to 1. Repeat this process to obtain 2-shell, 3-shell, $\ldots$ , and so on. The decomposition process will continue until there are no more nodes in the network.

The eigenvector centrality^13^ (EC) of node *i* is measured by

2 $$\begin{aligned} x(i)=c\sum _{j = 1}^{N}a_{ij}x(j), \end{aligned}$$

where *c* is a constant, generally speaking, *c* is set to the reciprocal of the largest eigenvalue of *A*.

The betweenness centrality^14^ (BC) of node *i* is measured by

3 $$\begin{aligned} BC(i)=\sum _{s\ne {i},s\ne {t},i\ne {t}}\frac{g_{st}(i)}{g_{st}}, \end{aligned}$$

where $g_{st}$ is the number of shortest paths between node *s* and node *t*, and $g_{st}(i)$ is the number of shortest paths via node *i* between node *s* and node *t*.

The closeness centrality^15^ (CC) of node *i* is measured by

4 $$\begin{aligned} CC(i)=\frac{N-1}{\sum \limits _{j\ne i} d(i,j)}, \end{aligned}$$

where *d*(*i*, *j*) is the shortest distance from node *i* to node *j*.

The gravity centrality^18^ (GC) of node *i* is measured by

5 $$\begin{aligned} GC(i)=\sum _{j\in \psi _i}\frac{k_s(i)k_s(j)}{d^{2}(i,j)}, \end{aligned}$$

where $k_s(i)$ is the *k*-shell value of node *i*, and $\psi _i$ is the neighborhood set whose distance to node *i* is not greater than 3.

An extended version of GC, denoted by GC+, GC+ of node *i* is measured by

6 $$\begin{aligned} GC+(i)=\sum _{j\in \Lambda _{i}}GC(j), \end{aligned}$$

where $\Lambda _{i}$ is the neighborhood set whose distance to node *i* equals to 1.

The improved gravity centrality^19^ (IGC) of node *i* is measured by

7 $$\begin{aligned} IGC(i)=\sum _{j\in \psi _i}\frac{k_s(i)k(j)}{d^{2}(i,j)}. \end{aligned}$$

An extended version of IGC, denoted by IGC+, IGC+ of node *i* is measured by

8 $$\begin{aligned} IGC+(i)=\sum _{j\in \Lambda _{i}}IGC(j). \end{aligned}$$

The local gravity model^20^ (LGM) of node *i* is measured by

9 $$\begin{aligned} LGM(i)=\sum _{d(i,j)\le R,j\ne {i}}\frac{k(i)k(j)}{d^{2}(i,j)}, \end{aligned}$$

where *R* is the truncation radius, and the optimal truncation radius $R^*$ can be estimated by

10 $$\begin{aligned} R^*\approx \frac{1}{2}\left\langle d \right\rangle , \end{aligned}$$

where $\left\langle d \right\rangle$ is the average distance of the network.

### The SIR model

The SIR model^31^ initially considers all the nodes as in the susceptible (S) state except the source node in the infected (I) state. At each time step, each infected node can infect its susceptible neighbors with probability $\beta$. Then, each infected node enters the recovered (R) state with probability $\lambda$. The propagation process continues until there are no more nodes in the infected state. The influence of node *i* can be estimated by

11 $$\begin{aligned} F(i) = N_{r}/N, \end{aligned}$$

where $N_{r}$ is the number of recovered nodes when dynamic process achieves steady state. For simplicity, $\lambda$ is set to 1, then the corresponding epidemic threshold^32^ can be calculated by

12 $$\begin{aligned} \beta _c\approx \frac{\left\langle k \right\rangle }{\left\langle k^{2} \right\rangle -\left\langle k \right\rangle }, \end{aligned}$$

where $\left\langle k \right\rangle$ is the average degree, and $\left\langle k^{2} \right\rangle$ is the second-order moment of the degree distribution.

### The Kendall’s Tau

The Kendall’s Tau^33^ is an index describing the strength of correlation between two sequences. Denote $$X=(x_1, x_2, \ldots ,x_N)$$ and $$Y=(y_1, y_2, \ldots , y_N)$$ are two sequences with *N* elements. For any pair of two-tuples $$(x_i,y_i)$$ and $$(x_j,y_j)$$ $$(i\ne j)$$, if both $$x_i>x_j$$ and $$y_i>y_j$$ or both $$x_i<x_j $$ and $$y_i<y_j$$, the pair is concordant. If both $$x_i>x_j$$ and $$y_i<y_j$$ or both $$x_i<x_j$$ and $$y_i>y_j$$, the pair is discordant. If $$x_i=x_j$$ or $$y_i=y_j$$, the pair is neither concordant nor discordant. The Kendall’s Tau of *X* and *Y* can be calculated by

13 $$\begin{aligned} \tau =\frac{2(n_+-n_-)}{N(N-1)}, \end{aligned}$$

where $n_+$ is the number of concordant pairs, and $n_-$ is the number of discordant pairs.

### The monotonicity

The monotonicity^34^ *M* of ranking list *L* is used to quantitatively measure the resolution of different indices, and it can be calculated by

14 $$\begin{aligned} M(L)=[1-\frac{\sum _{r\in L}U_{r}(U_{r}-1)}{U(U-1))}]^{^{2}}, \end{aligned}$$

where *U* is the size of *L*, and $U_{r}$ is the number of ties with the same rank *r*.

## Results

### Algorithms

According to previous studies, the degree value of a node indicates the number of its neighbors, the *k*-shell value of a node reflects where it locates in the network, the eigenvector centrality value of a node can reflect both the number of its neighbors and the influence of each neighbor, and the distance between two nodes can describe the path information. Individually speaking, nodes with large degree value, *k*-shell value and eigenvector centrality value are likely to be more influential. Furthermore, a node is of higher impacts on nearby nodes. According to the above issues and inspired by the gravity law, we regard the sum of degree value, *k*-shell value and eigenvector centrality value of a node as its mass, and the shortest distance between two nodes as their distance. Therefore, the influence of node *i* can be estimated as

15 $$\begin{aligned} MCGM(i)=\sum _{d(i,j)\le R, j\ne {i}}\frac{(k(i)+k_s(i)+x(i))(k(j)+k_s(j)+x(j))}{d^{2}(i,j)}. \end{aligned}$$

Such method is named as multi-characteristics gravity model (MCGM) as it considers multi-characteristics of nodes and adopts the gravity law.

It is not difficult to find that these three indices (DC, KS, EC) are not in the same order of magnitude, so normalization is required. As a result, Eq. (15) can be rewritten as

16 $$\begin{aligned} MCGM(i)=\sum _{d(i,j)\le R, j\ne {i}}\frac{(\frac{k(i)}{k_{max}}+\frac{k_{s}(i)}{k_{s{max}}}+\frac{x(i)}{x_{max}})(\frac{k(j)}{k_{max}}+\frac{k_{s}(j)}{k_{s{max}}}+\frac{x(j)}{x_{max}})}{d^{2}(i,j)}, \end{aligned}$$

where $k_{max}$, $k_{s{max}}$ and $x_{max}$ denote the maximum of degree value, *k*-shell value and eigenvector centrality value, respectively.

However, since the *k*-shell index has smaller value space, the normalized *k*-shell index is still larger than the other two indices. Therefore, it is necessary to lower the impact of the *k*-shell index. Given an index, due to the scale-free property of networks, the index values of most nodes are relatively small. Therefore, the index with larger value space generally has a smaller ratio between the median and the maximum. In our model, it is obvious that the value space of degree centrality and eigenvector centrality is larger than that of *k*-shell index. In view of this, we can lower the impact of *k*-shell index by

17 $$\begin{aligned} \alpha =\frac{max{\left\{ {\frac{k_{mid}}{k_{max}},\frac{x_{mid}}{x_{max}}}\right\} }}{\frac{k_{s{mid}}}{k_{s{max}}}}, \end{aligned}$$

where $k_{mid}$, $k_{s{mid}}$ and $x_{mid}$ denote the median of degree value, *k*-shell value and eigenvector centrality value, respectively. The purpose of taking the maximum value of ${\left\{ {\frac{k_{mid}}{k_{max}},\frac{x_{mid}}{x_{max}}}\right\} }$ is to prevent the function of *k*-shell index from being excessively weakened.

Finally, Eq. (15) can be rewritten as

18 $$\begin{aligned} MCGM(i)=\sum _{d(i,j)\le R, j\ne {i}}\frac{(\frac{k(i)}{k_{max}}+\frac{\alpha k_{s}(i)}{k_{s{max}}}+\frac{x(i)}{x_{max}})(\frac{k(j)}{k_{max}}+\frac{\alpha k_{s}(j)}{k_{s{max}}}+\frac{x(j)}{x_{max}})}{d^{2}(i,j)}. \end{aligned}$$

**Figure 1:**
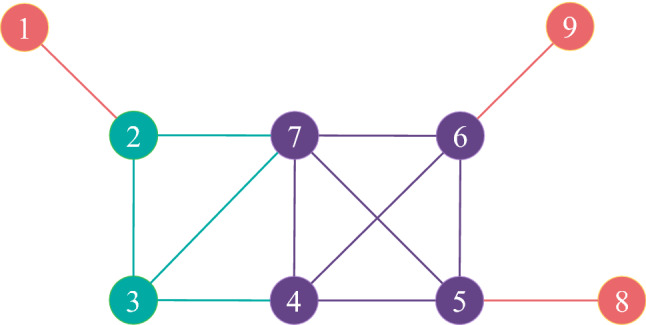
A toy network. The red nodes are in 1-shell, the green nodes are in 2-shell and the purple nodes are in 3-shell.

The Algorithmic description of MCGM is provided in Algorithm 1. We take a toy network shown in Fig. 1 to illustrate the calculation process of Algorithm 1.
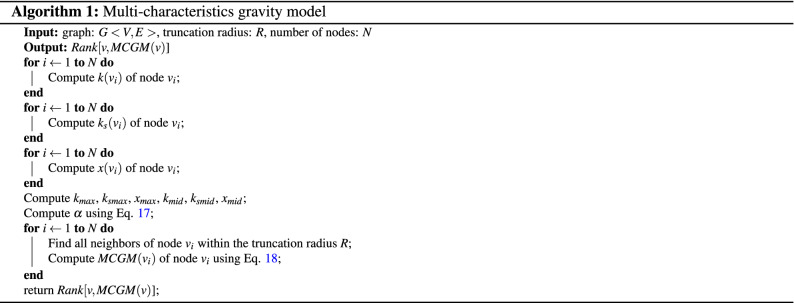

Firstly, calculate the degree value, *k*-shell value and eigenvector centrality value of each node in the toy network, the results are shown in Table 1.

**Table 1:** The degree value, *k*-shell value and eigenvector centrality value of each node in the toy network.

Node	DC	KS	EC
1	1	1	0.0259
2	3	2	0.0943
3	3	2	0.1256
4	4	3	0.1714
5	4	3	0.1534
6	4	3	0.1534
7	5	3	0.1917
8	1	1	0.0421
9	1	1	0.0421

Secondly, calculate $k_{max}=5$, $k_{s{max}}=3$, $x_{max}=0.1917$, $k_{mid}=3$, $k_{s{mid}}=2$ and $x_{mid}=0.1256$, furthermore, calculate $\alpha =0.9827$.

Finally, the result of MCGM with $R=2$ of the toy network is shown in Table 2. Take node 3 as an example, the 1-order neighbors of node 3 are node 2, node 4 and node 7, the 2-order neighbors of node 3 are node 1, node 5 and node 6, so $MCGM(3)=16.9320$.

**Table 2:** The result of MCGM with $R=2$ of the toy network.

Node	1-order neighbors	2-order neighbors	MCGM
1	2	3,7	1.9679
2	1,3,7	4,5,6	13.1293
3	2,4,7	1,5,6	16.9320
4	3,5,6,7	2,8,9	29.0955
5	4,6,7,8	2,3,9	26.0652
6	4,5,7,9	2,3,8	26.0652
7	2,3,4,5,6	1,8,9	35.9099
8	5	4,6,7	3.4704
9	6	4,5,7	3.4704

### Data description

In this paper, we apply ten real networks from six fields to test the performance of MCGM, including one transportation network (USAir^35^), one communication network (Email^36^), one infrastructure network (Power^37^), one technological network (Router^38^), two collaboration networks (Jazz^39^ and NS^40^) and four social networks (PB^41^, Facebook^42^, WV^43^ and Sex^44^). Table 3 shows these networks’ topological features, including the number of nodes, the number of links, the average degree, the average distance, the clustering coefficient^37^, denoted by *C*, the assortative coefficient^45^, denoted by *r*, the degree heterogeneity^46^, denoted by *H*, and the epidemic threshold^32^ of SIR model^31^.

**Table 3:** The topological features of ten real networks.

Networks	*N*	*M*	$\langle k \rangle$	$\langle d \rangle$	*C*	*r*	*H*	$\beta _c$
USAir	332	2126	12.8072	2.7381	0.7494	− 0.2079	3.4639	0.0231
Email	1133	5451	9.6222	3.6060	0.2540	0.0782	1.9421	0.0565
Power	4941	6594	2.6691	18.9892	0.1065	0.0035	1.4504	0.3483
Router	5022	6258	2.4922	6.4488	0.0329	− 0.1384	5.5031	0.0786
Jazz	198	2742	27.6970	2.2350	0.6334	0.0202	1.3951	0.0266
NS	379	914	4.8232	6.0419	0.7981	− 0.0817	1.6630	0.1424
PB	1222	16714	27.3552	2.7375	0.3600	− 0.2213	2.9707	0.0125
Facebook	4039	88234	43.6910	3.6925	0.6170	0.0636	2.4392	0.0095
WV	7066	100736	28.5129	3.2475	0.2090	− 0.0833	5.0992	0.0069
Sex	15810	38540	4.8754	5.7846	0.0000	− 0.1145	5.8276	0.0365

### Empirical results

**Figure 2:**
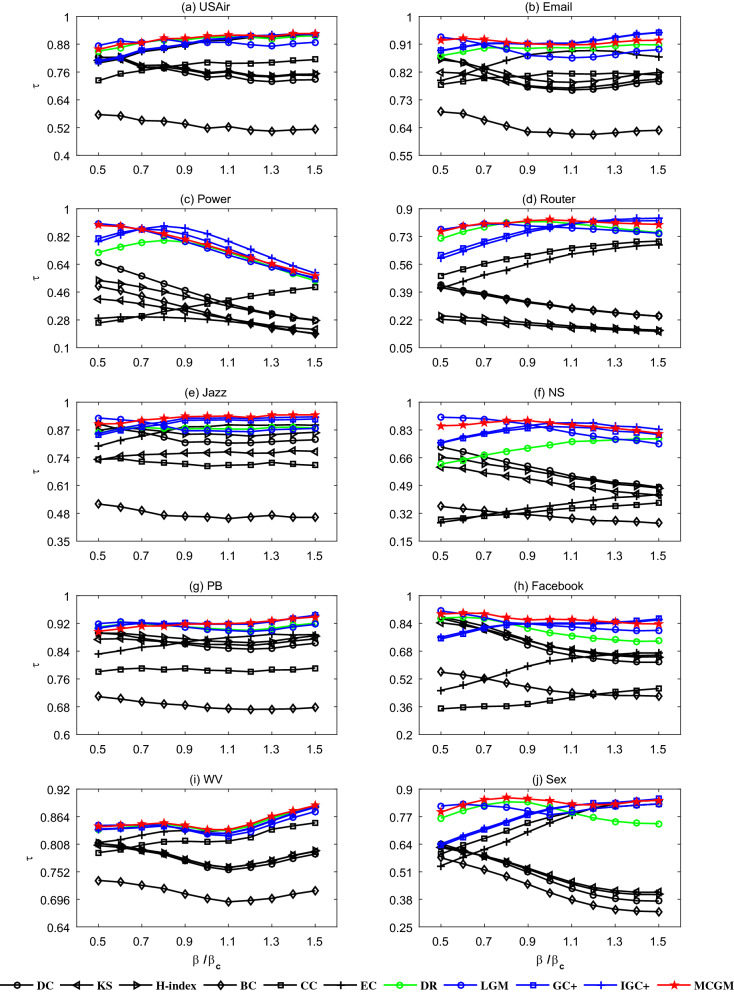
The algorithms’ accuracies measured by Kendall’s Tau for different $\beta$. The six classic algorithms (DC, H-index, KS, EC, BC and CC) are represented by black symbols, DR is represented by green symbols, the typical algorithms based on the gravity law (GC+, IGC+ and LGM) are represented by blue symbols, MCGM is represented by red symbols.

Based on the above real networks, the well-known SIR model^31^ is used to compare the influential rankings produced by algorithms and simulations. Given the network and the transmission probability $\beta$, in order to guarantee the reliability of the results, 1000 independent realizations are executed and averaged to obtain the standard ranking of the influence of nodes (see details about SIR model in Preliminaries). In each realization, every node is selected once as the seed once. We apply the Kendall’s Tau ($\tau$) between the standard ranking and the ranking produced by the algorithm to measure the accuracy of an algorithm. Since $\tau \in \left[ -1,1\right]$, the closer the $\tau$ is to 1, the better the performance of the algorithm. The benchmark algorithms include degree centrality^10^ (DC), H-index^11^, *k*-shell decomposition method^12^ (KS), eigenvector centrality^13^ (EC), betweenness centrality^14^ (BC), closeness centrality^15^ (CC), DynamicRank^47^ (DR), the extended version of gravity centrality^18^ (GC+), the extended version of improved gravity centrality^19^ (IGC+) and local gravity model^20^ (LGM). Table 4 compares the accuracies of MCGM and the ten benchmark algorithms for $\beta =\beta _c$. Furthermore, the accuracies of different $\beta$ values (not too far from $\beta _c$) are shown in Fig. 2.

**Table 4:** The algorithms’ accuracies of MCGM and the benchmark algorithms measured by Kendall’s Tau for $\beta =\beta _c$.

Networks	DC	H-index	KS	EC	BC	CC	DR	GC+	IGC+	LGM	MCGM
USAir	0.7370	0.7568	0.7529	0.8946	0.5171	0.8027	0.9096	0.8985	0.9006	0.8875	**0.9145**
Email	0.7653	0.7883	0.7702	0.8832	0.6243	0.8163	0.8991	0.9119	**0.9133**	0.8697	0.9091
Power	0.4264	0.4009	0.3122	0.2818	0.3254	0.3838	0.7570	0.7906	**0.8387**	0.7442	0.7639
Router	0.3139	0.1928	0.1810	0.5924	0.3096	0.6383	0.8215	0.7896	0.7823	0.7894	**0.8324**
Jazz	0.8150	0.8513	0.7638	0.8854	0.4641	0.7008	0.8761	0.9158	0.9244	0.8666	**0.9333**
NS	0.5790	0.5610	0.5106	0.3660	0.3003	0.3397	0.7377	0.8511	0.8722	0.8372	**0.8736**
PB	0.8524	0.8694	0.8595	0.8738	0.6771	0.7852	0.9060	**0.9189**	0.9176	0.9030	0.9184
Facebook	0.6798	0.7066	0.7075	0.6226	0.4529	0.3940	0.7865	0.8414	0.8372	0.8275	**0.8639**
WV	0.7619	0.7662	0.7657	0.8334	0.6978	0.8127	0.8360	0.8298	0.8305	0.8276	**0.8379**
Sex	0.4664	0.4855	0.4925	0.7404	0.4118	0.7677	0.8139	0.8038	0.8076	0.7789	**0.8448**

The parameters in the related algorithms (i.e., LGM and MCGM) are adjusted to their optimal values subject to the largest $\tau$, that is, we need to search the optimal truncation radius which can maximize $\tau$ by traversing the truncation radius. Obviously, searching the optimal truncation radius in this way is very time-consuming, fortunately, in subsequent experiments, we find that MCGM still performs very competitively even if the truncation radius is just set to 2. For each network, the best algorithm is emphasized by bold.

As shown in Table 4, the methods based on gravity law (GC+, IGC+, LGM and MCGM) show great advantages over the classic methods (DC, H-index, KS, EC, BC, CC), especially in Power, Router and NS, the advantage of the methods based on gravity law are extremely obvious. Notice that, except the above three networks, the performance of EC is significantly better than other classic methods, and even performs competitively in comparison with the methods based on gravity law, which indirectly shows that the stability of the method based on the gravity law is better and their performance will not decline precipitously due to the differences of networks. Furthermore, for the methods based on gravity law, MCGM generally performs best since it effectively considers more characteristics of nodes. As shown in Fig. 2, MCGM still performs very competitively compared with the ten benchmark algorithms for different $\beta$ not too far from $\beta _c$, suggesting the robustness of our findings.

**Figure 3:**
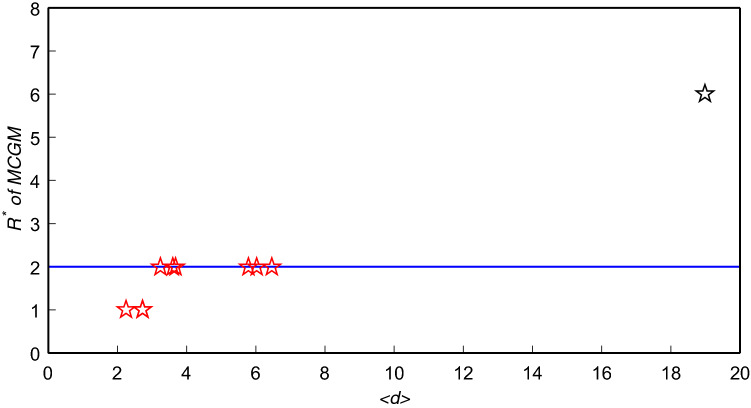
The $R^*$ of MCGM for $\beta =\beta _c$. Ten pentagrams represent ten networks and the blue line is $R=2$. The $R^*$ of MCGM in USAir, Jazz and PB is 1, the $R^*$ of MCGM in Email, Router, NS, Facebook, WV and Sex is 2, and the $R^*$ of MCGM in Power is 6.

Figure 3 shows the optimal truncation radius of MCGM in the ten real networks. It is not difficult to find that the optimal truncation radius of most networks is concentrated at $R=2$. Therefore, we may simply set $R=2$ to test the performance of MCGM. Table 5 compares the accuracies of MCGM with $R=2$ and the benchmark algorithms.

**Table 5:** The algorithms’ accuracies of MCGM with $R=2$ and the benchmark algorithms measured by Kendall’s Tau for $\beta =\beta _c$.

Networks	DC	H-index	KS	EC	BC	CC	DR	GC+	IGC+	LGM	MCGM ($R=2$)
USAir	0.7370	0.7568	0.7529	0.8946	0.5171	0.8027	**0.9096**	0.8985	0.9006	0.8875	0.9092
Email	0.7653	0.7883	0.7702	0.8832	0.6243	0.8163	0.8991	0.9119	**0.9133**	0.8697	0.9091
Power	0.4264	0.4009	0.3122	0.2818	0.3254	0.3838	0.7570	0.7906	**0.8387**	0.7442	0.6616
Router	0.3139	0.1928	0.1810	0.5924	0.3096	0.6383	0.8215	0.7896	0.7823	0.7894	**0.8324**
Jazz	0.8150	0.8513	0.7638	0.8854	0.4641	0.7008	0.8761	0.9158	0.9244	0.8666	**0.9255**
NS	0.5790	0.5610	0.5106	0.3660	0.3003	0.3397	0.7377	0.8511	0.8722	0.8372	**0.8736**
PB	0.8524	0.8694	0.8595	0.8738	0.6771	0.7852	0.9060	**0.9189**	0.9176	0.9030	0.9123
Facebook	0.6798	0.7066	0.7075	0.6226	0.4529	0.3940	0.7865	0.8414	0.8372	0.8275	**0.8639**
WV	0.7619	0.7662	0.7657	0.8334	0.6978	0.8127	0.8360	0.8298	0.8305	0.8276	**0.8379**
Sex	0.4664	0.4855	0.4925	0.7404	0.4118	0.7677	0.8139	0.8038	0.8076	0.7789	**0.8448**

For each network, the best algorithm is emphasized by bold.

As shown in Table 5, MCGM with $R=2$ generally performs best in comparison with the benchmark algorithms, it still obtains the best results in six of the ten real networks. Since the optimal truncation radius approximately scales linearly with the average distance^20^, if the average distance of the network is relatively large, setting $R=2$ will have a significant impact on the performance of MCGM, such as Power whose average distance is 18.9892. Fortunately, most real networks have small-world property, $R^*$ tends to be small in most cases.

Furthermore, we need to compare MCGM and MCGM without normalization to illustrate the importance of normalization. Table 6 compares the accuracies of MCGM using Eq. (15), MCGM using Eq. (16) and MCGM using Eq. (18). As shown in Table 6, MCGM has been gradually improved by normalization, suggesting the importance of normalization and the effectiveness of our normalization strategy.

**Table 6:** The algorithms’ accuracies of MCGM using Eq. (15), MCGM using Eq. (16) and MCGM using Eq. (18) measured by Kendall’s Tau for $\beta =\beta _c$.

Networks	MCGM (Eq. 15)	MCGM (Eq. 16)	MCGM (Eq. 18)
USAir	0.8946	0.9060	**0.9145**
Email	0.8782	0.8986	**0.9091**
Power	0.7557	0.7569	**0.7639**
Router	0.7992	0.7983	**0.8324**
Jazz	0.8888	0.9212	**0.9333**
NS	0.8428	0.8710	**0.8736**
PB	0.9047	0.9110	**0.9184**
Facebook	0.8381	0.8547	**0.8639**
WV	0.8299	0.8341	**0.8379**
Sex	0.7877	0.7996	**0.8448**

The parameters are adjusted to their optimal values subject to the largest $\tau$. For each network, the best algorithm is emphasized by bold.

Finally, we apply the monotonicity^34^ to measure the resolution of different algorithms. As shown in Table 7, MCGM generally performs best even if it only considers 1-order neighbors or 2-order neighbors in most cases. The results reported in Table 7 demonstrate MCGM is a remarkably high-resolution algorithm.

**Table 7:** The monotonicity of different algorithms. The parameters in the related algorithms (i.e., LGM and MCGM) are adjusted to their optimal values subject to the largest $\tau$.

Networks	DC	H-index	KS	EC	BC	CC	DR	GC+	IGC+	LGM	MCGM
USAir	0.8586	0.8355	0.8114	0.9951	0.6970	0.9892	0.9951	**0.9951**	0.9951	0.9933	0.9951
Email	0.8874	0.8583	0.8088	0.9999	0.9400	0.9988	0.9999	0.9999	**0.9999**	0.9998	0.9999
Power	0.5927	0.3930	0.2460	0.9999	0.8314	0.9998	0.9962	0.9996	0.9997	0.9999	**0.9999**
Router	0.2886	0.0876	0.0691	0.9964	0.2985	0.9961	0.9956	0.9965	0.9965	0.9964	**0.9966**
Jazz	0.9659	0.9383	0.7944	0.9994	0.9885	0.9878	0.9993	**0.9995**	0.9993	0.9991	0.9994
NS	0.7642	0.6825	0.6421	0.9955	0.3388	0.9928	0.9950	0.9954	**0.9956**	0.9933	0.9955
PB	0.9328	0.9268	0.9064	0.9993	0.9489	0.9980	0.9993	0.9993	0.9993	0.9991	**0.9993**
Facebook	0.9739	0.9665	0.9419	0.9999	0.9855	0.9967	0.9999	0.9999	0.9999	0.9999	**0.9999**
WV	0.7761	0.7732	0.7673	0.9996	0.7704	0.9994	0.9996	0.9996	0.9996	0.9996	**0.9996**
Sex	0.6002	0.5457	0.5288	0.9997	0.6757	0.9996	0.9996	0.9997	0.9997	0.9997	**0.9997**

For each network, the best algorithm is emphasized by bold.

### Computational complexity

The computational complexity of the methods used in this paper is shown in Table 8. The computational complexity of DC, KS and EC is *O*(*N*), *O*(*M*) and $O(N+M)$, respectively. Therefore, it is obvious that the part with the highest computational complexity of MCGM is computing the *R*-order neighbors of each node, it needs $N \left\langle k \right\rangle ^{R}$ times operations. Hence the computational complexity of MCGM is $O(N \left\langle k \right\rangle ^{R})$. Since most real networks have small-world property, $R^*=2$ in most cases (see Fig. 3), so the computational complexity of MCGM is generally not more than $O(N \left\langle k \right\rangle ^2)$, where $\left\langle k \right\rangle \ll N$.

**Table 8:** The computational complexity of MCGM and the benchmark algorithms.

Methods	Topology	Complexity
DC	Local	*O*(*N*)
H-index	Semi-local	$O(N+M)$
KS	Global	*O*(*M*)
EC	Global	$O(N+M)$
BC	Global	$O(NM+N^{2}logN)$
CC	Global	$O(NM+N^{2}logN)$
DR	Semi-local	$O(N\langle k \rangle ^{3})$
GC+	Global	$O(N\langle k \rangle ^{3})$
IGC+	Global	$O(N\langle k \rangle ^{3})$
LGM	Semi-local	$O(N\langle k \rangle ^{R})$
MCGM	Global	$O(N\langle k \rangle ^{R})$

## Discussion

In summary, we propose a novel gravity model that effectively integrates multi-characteristics of nodes, named as multi-characteristics gravity model (MCGM). The number of neighbors, the influence of neighbors, the location of nodes, and the path information between nodes are all taken into consideration in our model. In addition, we propose a normalization strategy to solve the problem that different indices are not in the same order of magnitude, Table 6 suggests the importance of normalization and the effectiveness of our normalization strategy. Compared with well-known state-of-the-art methods, empirical analyses of the SIR spreading dynamics on ten real networks suggest that our model always performs very competitively, as shown in Table 4.

However, MCGM needs to find the optimal truncation radius by traversing the truncation radius and it is very time-consuming. Fortunately, the optimal truncation radius approximately scales linearly with the average distance^20^, and most real networks have small-world property^37,48^, so even if the truncation radius is just set to 2, MCGM still performs very competitively in most cases, as shown in Table 5. Therefore, without increasing the computational complexity, MCGM effectively considers more characteristics of nodes and obtains more accurate results.

Although the computational complexity of MCGM is not high, it needs the global topological structure, same as GC+ and IGC+. While LGM can work under the case where the global topology is not known. As a result, our suggestions for practical use are as follows: if the network’s global topology is known, apply MCGM and set *R* to 2, otherwise, apply LGM and set *R* to 2 or 3.

Of course, there are still some potential problems in the future. First of all, the gravity law is symmetrical, but due to the different effects of different nodes or the inherent asymmetry of dynamics^49,50^, an asymmetric form of the gravity law may be relevant. Secondly, in weighted complex networks, the heterogeneity of links greatly changes nodes’ importance^51^, a weighted form of the gravity law may be relevant. Finally, in order to establish a unified research framework, a unified gravity model is needed to be proposed. Although GC+, IGC+ and LGM are proposed from different perspectives, a unified form of expression exists. We propose a rough model which intends to start further discussion on this issue. The rough unified gravity model is described as

19 $$\begin{aligned} UGM(i)=\sum _{d(i,j)\le R,j\ne {i}}\frac{(ak(i)+(1-a)k_{s}(i))(bk(j)+(1-b)k_{s}(j))}{d(i,j)^{2}}, \end{aligned}$$

where *a* and *b* are adjustable parameters. If $a=1$ and $b=1$, the unified gravity model degenerates to LGM. If $a=0$ and $b=0$, the unified gravity model degenerates to GC (GC+ can be obtained by Eq. (6)). If $a=0$ and $b=1$, the unified gravity model degenerates to IGC (IGC+ can be obtained by Eq. (8)).

## Data Availability

All relevant data are available at https://github.com/MLIF/Network-Data.
